# Regional challenges in acknowledging and battling obstetric violence

**DOI:** 10.18332/ejm/202344

**Published:** 2025-04-09

**Authors:** Alieke De Roon-Immerzeel, Maaike van Rijn, Marieke Smith, Suzanne M. Thompson

**Affiliations:** 1 Royal Dutch Organization of Midwives (KNOV), Utrecht, Netherlands

**Keywords:** obstetric violence

In March 2024, a joint position statement, ‘Substandard and Respectful Care – Because Words Matter’^1^ was published in the European Journal of Obstetrics and Gynaecology. This position paper was co-authored by the board members of the European Midwives Association (EMA), in collaboration with the European Association of Perinatal Medicine (EAPM) and the European Board and College of Obstetricians and Gynaecologists (EBCOG). The publication of the joint position statement attracted international attention^2-4^. In this guest editorial, the Royal Dutch Association of Midwives (KNOV) would like to highlight a number of issues with the current statement (written in collaboration with obstetric violence scholar, Rodante van der Waal).

Since the early 2000s, the term obstetric violence has been globally recognized as a form of gender-based violence and violence against women. In various countries, it has been taken up in the law as a form of gender-based violence^5^. Both the European Commission^6^, the European Parliament^7^, and the United Nations^8^ have recognized both the term obstetric violence as well as the major occurrence of obstetric violence in Europe, in extensive reports. The refusal to recognize the term by the main professional organizations involved, goes against a global emancipatory movement that fights for women’s rights and dignity.

The KNOV and its members, midwives in the Netherlands, and Dutch maternity consumer organizations, are of the opinion that replacing obstetric violence with other terms, such as ‘substandard care’, diminishes parent’s experiences and perceptions of their care, and obstructs midwifery care, in at least four ways: 1) by not taking seriously a form of gender-based violence and violence against women, thus contributing to the continuous dismissal and normalization of a form of violence against women; 2) by negating a whole body of scholarly research that has been building over the years; 3) by going fundamentally against ‘being with-women’ or ‘woman-centered’ care; and 4) by taking a defensive stance against the people we, as midwives, are supposed to be in solidarity with, hence violating mothers’ trust, and the good name of the profession of midwifery. Let us take a closer look at these four points.

Firstly, when marginalized groups articulate the oppression that they experience, it is a common reaction to deny their articulation. This denial is part of the very same structure of oppression: it is that which normalizes the oppression and violence suffered. In scholarly literature, this is known as ‘epistemic injustice’ and ‘gaslighting’, concepts that point out the double harm of suffering violence and not being heard, believed, or taken seriously when trying to articulate it. Mothers did not call their experiences ‘substandard care’, they named it violence because this captured their experience best.

Secondly, refusing to use the term obstetric violence invisibilizes an extensive and growing body of scholarly work^8-12^. Not only since this work cannot be found when refusing to use the right keyword, but also since experts have extensively analyzed why parents use the term ‘violence’ – this is not a random choice. With ‘violence’ a form of systemic, institutionalized, sexist violence is meant, making it comparable to other forms of systemic violence, such as racism and misogyny, and making it possible to understand it within a theoretical framework of gender-based violence and violence against women stemming from feminist sociology and critical theory. It is highly unscientific to change this terminology to ‘substandard care’, since this is a completely different term, rooted in a fundamentally different scholarly tradition, namely epidemiology, with no ties to feminist politics and theory, and devoid of any remains of mother’s own lived experiences.

Thirdly, specifically in the field of midwifery, not taking mothers’ articulation of their experiences of oppression seriously, can have dire consequences for the philosophy and practice of midwifery, since midwifery, alternatively to obstetrics, is founded upon solidarity with women, its core principle is ‘being with-women’. Especially for a midwifery organization, then, it is highly irresponsible not to side with women when it comes to this matter: it damages the foundation of our profession and the core of midwifery philosophy. According to the influential care ethicist Joan Tronto^13^, whether something is care or not, can only be determined by the people who are the recipients of care. If our care is experienced as violence, it cannot be considered as care and also not ‘substandard’ care. As professional organizations in the field of reproductive care who are committed to woman-centered care, we must do the right thing by the mothers and parents we aim to care for, through engaging with their experiences and the vast field of research and activism that aims to amplify and analyze their experiences, so that we can reflect, be self-critical, and change our practices for the better. If we wish to continue to practice by the philosophy of being with-women and woman-centered care, we must take the voices and experiences of women seriously and we can only do that by listening to them and being open to criticism, so we can improve our practice.

Fourthly, turning against the term obstetric violence would mean turning against the people we care for. We cannot have it both ways: we cannot be the representatives of mothers and deny their experiences when these same mothers say they are victims of violence. We cannot be the allies of pregnant women, and at the same time dismiss the harm they say they suffer at our hands. If midwifery wishes to continue to be the voice of women and fight for rights and dignity in childbirth, we cannot afford to be on the wrong side on one of the most urgent problems in maternity care today. This breaks a relation of trust that took decades to rebuilt and is still hard-won today. Midwives could be the allies of pregnant women in the fight against obstetric violence and even be the alternative to that violence, but only if we indeed are brave enough to stand against it.

The KNOV, in representing its members and as advocates of the people we serve, chooses to acknowledge and accept the language that parents use to voice their experiences at the hands of caregivers during childbirth. We do this because we too, understand that words matter.

## Data Availability

Data sharing is not applicable to this article as no new data were created.
